# Supplementary material for: BceAB-Type Antibiotic Resistance Transporters Appear To Act by Target Protection of Cell Wall Synthesis
Source: Antimicrob Agents Chemother. 2020 Feb 21;64(3):e02241-19. doi: 10.1128/AAC.02241-19 (PMC7038271; doi:10.1128/AAC.02241-19)
Supplement: Supplemental file 1 [file AAC.02241-19-s0001.pdf]

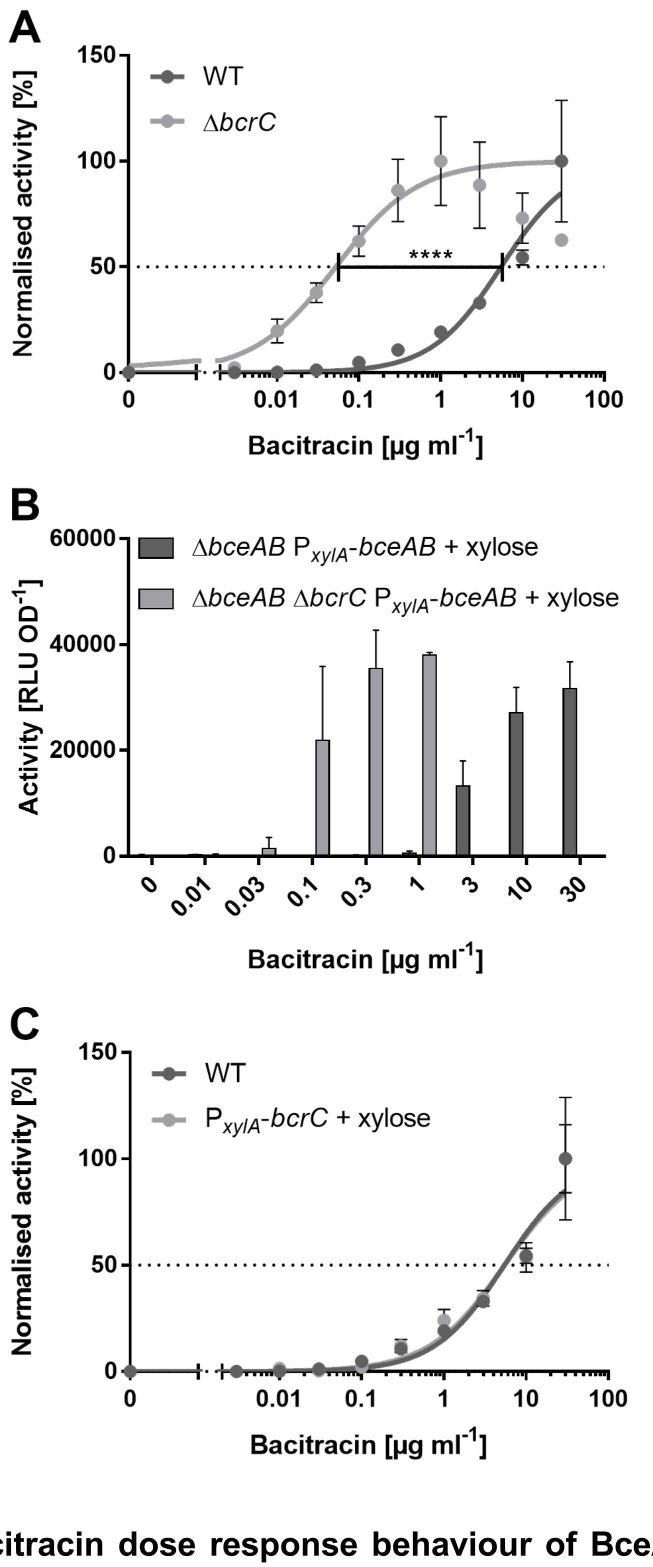

Figure S1: Bacitracin dose response behaviour of BceAB. A&C: Bacitracin dose response curves of BceAB activity were fitted on normalised data of the WT (SGB73) and Δ*bcrC* mutant (A, SGB649), or BcrC overproduction strain (C, SGB758). To obtain the best fit of experimental data a non-linear fit with variable slope was chosen. Statistical analyses of the log(EC_{50}) values using the in-build non-linear regression comparison of GraphPad Prism7 showed a significant difference between the WT and Δ*bcrC* mutant (****: $p < 0.0001$), but no difference between WT and BcrC overproduction strain (p = 0.73). **B:** BceAB activity was tested in wild-type (SGB218) and Δ*bcrC* strains (SGB677), in which BceAB production was uncoupled from its native regulation. Expression of *bceAB* was induced by addition of 0.2 % (w/v) xylose. All data are shown as mean ± standard deviation of at least three biological replicates.

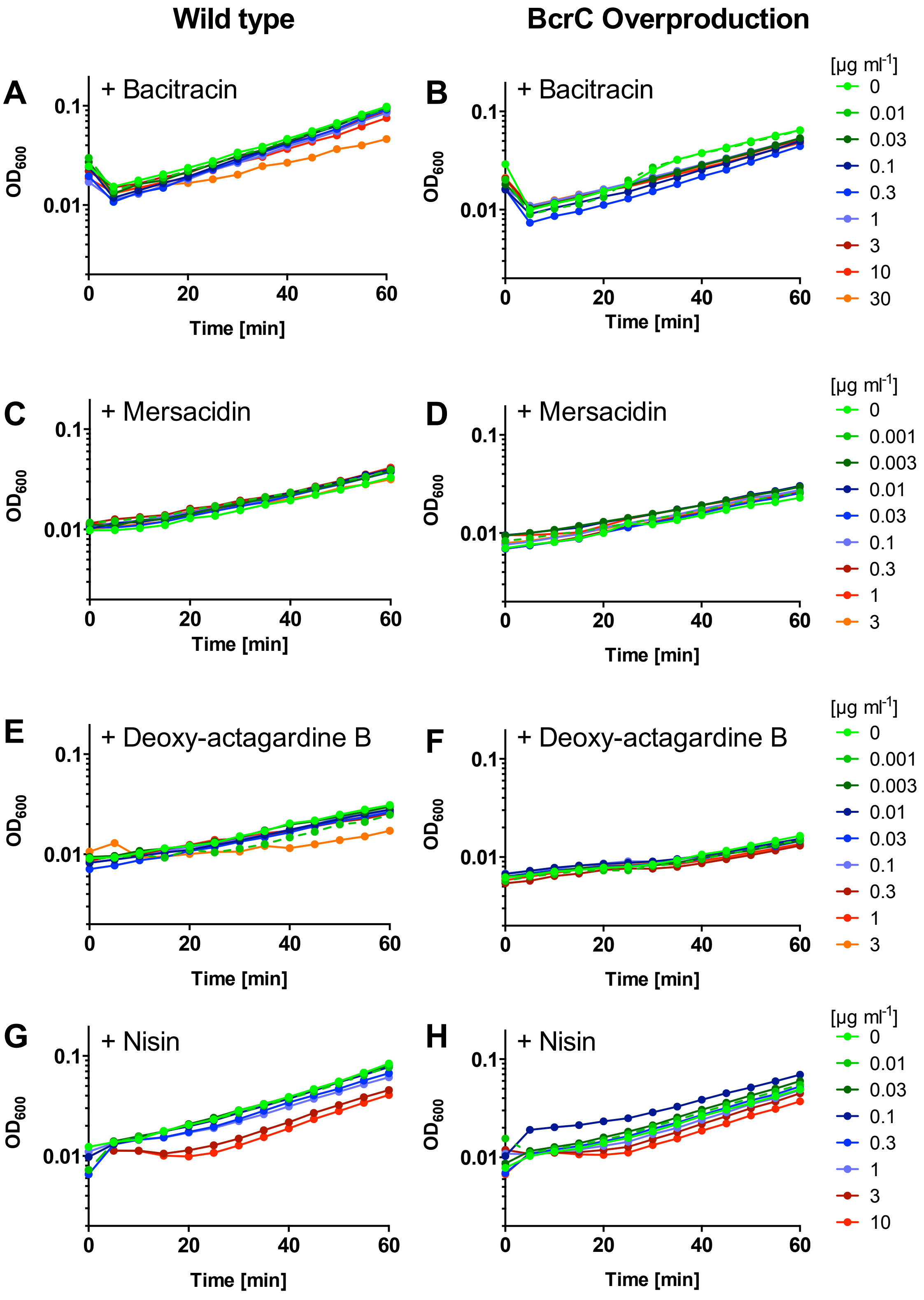

Figure S2: Growth curves following antibiotic exposure of the wild-type and BcrC overproduction strains. Exponentially growing cells of P_{bceA}-*lux* or P_{psdA}-*lux* reporter derivatives of the wild type (SGB73 or SGB74; left) or BcrC overproduction strain (SGB758 or SGB974; right) were challenged with different concentrations of bacitracin, mersacidin, deoxy-actagardine B and nisin (as indicated) at time point 0 min and OD_{600} was monitored over 60 min. Panels A, B, C, D, E and F show P_{bceA}-*lux* strains, panels G and H P_{psdA}-*lux* strains. Data shown are representative growth curves of at least three biological replicates.
